# Standardized Platelet Rich Plasma Injections for Osteoarthritis of the Knee

**DOI:** 10.7759/cureus.10900

**Published:** 2020-10-11

**Authors:** Cooper B Ehlers, Alex R Webb, Brian P McCormick, Trevor J Wyand, Neil Sarna, Kathryn Povey, Geoffrey Marano, Lawrence Schainker

**Affiliations:** 1 Orthopaedics, Georgetown University School of Medicine, Washington DC, USA; 2 Orthopaedics, Emory University School of Medicine, Atlanta, USA; 3 Orthopaedics, MedStar Union Memorial Hospital, Baltimore, USA; 4 Orthopaedic Surgery, Georgetown University School of Medicine, Washington DC, USA; 5 Emergency Medicine, Georgetown University School of Medicine, Washington DC, USA; 6 Orthopaedics, MedStar Georgetown University Hospital, Washington DC, USA; 7 Rheumatology, MedStar Georgetown University Hospital, Washington DC, USA

**Keywords:** platelet rich plasma, adipose derived stem cells, knee osteoarthritis, knee pain, orthopaedic surgery, rheumatology, orthobiologics

## Abstract

Objective

Platelet-rich plasma (PRP) and adipose-derived stem cells (ADSC) injections are non-surgical treatments for knee osteoarthritis (OA). The purpose of this study is to assess the effectiveness of serial PRP with or without ADSC injections in the treatment of refractory OA of the knee.

Design

Patients who failed to achieve pain relief with conventional non-surgical treatments, with Kellgren-Lawrence grade 3 or 4 knee OA, were recruited from a private outpatient clinic. Over 67 patients were elected to receive serial PRP injections and 22 patients were elected to receive an ADSC+PRP injection. These patients completed Western Ontario and McMaster Universities Arthritis Index (WOMAC) surveys prior to each treatment and at follow-up appointments. These surveys were retrospectively reviewed to assess changes in functional status and pain over time.

Results

Twenty-nine patients from the PRP group and eight patients in the ADSC+PRP group had adequate follow-up for inclusion in the analysis. The PRP group had an improvement in WOMAC scores by 34.30%, 60.2%, and 58.5% for patients reporting at 1-3, 4-6, and >6 months of follow-up. The ADSC+PRP group experienced an improvement of 51% at an average of 4.66 months of follow-up.

Conclusions

Serial PRP injections and a single ADSC+PRP injection yield improved and sustained functional outcome scores for patients with severe, refractory OA of the knee. Future studies should consider consistent orthobiologic preparation protocols to ensure reproducibility.

## Introduction

Osteoarthritis (OA) is a common and debilitating degenerative joint disease that affects more than 30 million Americans [1-3]. In the past 70 years, the prevalence of OA has doubled [4], and nearly 50% of adults aged 75 years and older experience OA of the knee [2]. OA is a leading cause of disability [2] and is rising globally [5]. OA of the hip and knee can be particularly debilitating, and the rates of primary knee and hip arthroplasty have risen concomitantly [6], and associated significant costs [2,6-8] are expected to grow.

However, there are other options available to patients seeking conservative management of their symptomatic OA. Injectable orthobiologics such as platelet-rich plasma (PRP) have shown mixed efficacy [9-12] with data indicating relative superiority over other injectables such as hyaluronic acid [10-12]. As biological warehouses for more than 300 different growth factors (GFs), chemokines, and proteins [13], it is believed that the high concentrations of platelets found in PRP improve symptoms of OA through a variety of direct and indirect mechanisms. PRP directly promotes bone and tissue regeneration most likely by exerting a chemotactic, proliferative, and differentiating effect on mesenchymal stem cells [14,15]. In vivo evidence has shown PRP’s ability to directly induce proliferation and chondrogenesis in chondrogenic progenitor cells [16]. Chemical mediators of these effects released by the platelets in PRP include hepatocyte GF, platelet-derived GF, insulin-like GF-1, among others [13,15]. PRP releasate also promotes the maintenance and protection of articular cartilage by releasing a complex mix of both pro- and anti-inflammatory mediators that interfere with the NFkB pathway and stimulate synovial fibroblasts to produce chondroprotective factors [13,17].

The purpose of this retrospective review of prospectively collected data is to assess the effectiveness of serial PRP injections in patients with refractory OA of the knee. We hypothesize that serial PRP injections will have improved functional outcome status of the patient cohort at each of the multiple points of follow-up.

## Materials and methods

Approval for this study was obtained from the Georgetown-MedStar Institutional Review Board. After the failure to achieve pain relief with conventional non-surgical treatments, 89 patients with Kellgren-Lawrence grade 3 or 4 knee OA elected to receive serial injections of either PRP (n = 67) or adipose-derived stem cell + PRP (ADSC + PRP) (n = 22). No randomization occurred during this treatment allocation process and no control group was available. Patients completed Western Ontario and McMaster Universities Arthritis Index (WOMAC) [18] score surveys prior to every treatment and at follow-up appointments. Patient surveys were retrospectively reviewed and assessed for changes over time. Exclusion criteria for receiving treatment included current smokers and those patients on vitamin-K antagonist anticoagulation therapy. Patients on low-dose aspirin and direct-acting oral anticoagulants were eligible for treatment.

PRP injections were collected and processed according to the Emcyte 60 mL PRP kit (EmCyte Corporation, Fort Myers, USA) and protocol. An Emcyte centrifuge was used to deliver red blood cells and polymorphonuclear free PRP containing 10-15% monocytes. This produced a PRP platelet count generally four times the serum baseline. Prior to PRP injections, supra-patellar effusion when present were aspirated, and the supra-patellar recess was lavaged with 10cc of sterile saline. PRP injections were delivered under ultrasound guidance utilizing the #22 Havel Echoblock Needle (Havels, Inc., Cincinnati, USA) with 4cc delivered to the suprapatellar recess, 2cc to the medial, and 2cc to the lateral subcapsular spaces. Subcapsular space injections included injections to the medial border of the medial meniscus as well as the lateral border of the lateral meniscus. The walls of the supra-patellar recess and subcapsular spaces were then fenestrated with the injecting needle to expose collagen and facilitate targeted aggregation of the PRP and platelet releasate.

Adipose harvest for ADSC injections utilized the U.S. Stem Cell Company’s abdominal fat liposuction protocol. Aspirated fat, however, was not processed with an enzyme, but rather was manually macerated by repeated passage through a 2.3 mm filter followed by repeated passage through a 1.2 mm filter. The macerated fat aspirate was then centrifuged, and supernatant oil and debris were removed. The remaining stem cell precursor rich fat was resuspended in sterile saline and recentrifuged for final supernatant removal. The washed fat was then injected intra-articularly under ultrasound guidance utilizing a 10cc syringe and #14 gauge needle. Injection ports were the suprapatellar recess, medial and lateral subcapsular zones to include the medial border of the medial meniscus, and the lateral border of the lateral meniscus. Each fat injection was followed by an injection of PRP in the same anatomic space through the same needle. In general, a total of 10cc of fat and 8cc of PRP was utilized per joint. Bandaid dressing was applied to skin puncture sites. A cold pack was then applied to the injected knee for 15 minutes. Patients were instructed to use acetaminophen 1000 mg as needed up to three times daily for post-procedure pain and advised to refrain from impact loading and deep knee bending activities for seven days. A physical therapy regimen was also recommended involving light quad and hamstring strengthening. The protocol involved 25 daily repetitions each of 5 lb quadricep extensions and 5 lb hamstring curls with a static, three-second hold at end concentric range of motion.

The data from the initial baseline survey and from each follow-up survey were analyzed with JMP Pro 14 software (SAS Institute, Inc., Cary, USA) via a matched-pairs analysis. In comparison to baseline data, comparisons of total Western Ontario and McMaster Universities Osteoarthritis Index (WOMAC) scores and its three subsections were made utilizing paired t-tests to determine significance with a confidence interval of 95% (alpha = 0.05).

## Results

Overall, 67 patients with osteoarthritis of the knee were treated in this study. Twenty-nine patients were determined to have an adequate follow-up for inclusion in statistical analysis. The patients had an average age of 70.49 years with a standard deviation of 10.25. The patients with adequate follow-up data include 12 patients who received treatment in their left knees, 13 patients who received treatment in their right knees, and 2 patients who received injections bilaterally. With the intention of administering the three follow-up surveys at three, six, and nine months after the initial injection, the average follow-up surveys were administered at 2.7, 5.8, and 10.3 months, respectively. The results are displayed in Table 1.

**Table 1:** Matched Analysis of WOMAC Scores SD: standard deviation, WOMAC: Western Ontario and McMaster Universities Osteoarthritis Index.

	Baseline		Following First Injection (n = 29; t = 3 months)				Following Second Injection (n = 18; t = 6 months)				Following Third Injection (n = 5; t = 9+ months)			
WOMAC	Mean	SD	Mean	SD	% Reduction from baseline	p-value	Mean	SD	% Reduction from baseline	p-value	Mean	SD	% Reduction from baseline	p-value
Total	39.73	18.25	25.62	16.85	34.3	0.0002	15.5	12.78	60.2	<0.0001	16.2	18.1	58.5	0.0433
Pain	7.93	3.73	5.10	3.55	35.7	0.0004	2.44	2.15	69.2	0.0001	2	2.35	74.8	0.0086
Stiffness	3.55	1.94	2.45	1.55	31.1	0.0083	1.67	1.78	53.1	0.0027	2.4	1.52	32.4	0.065
Function	27.5	13.7	18.07	12.31	34.3	0.0002	11.39	9.65	58.6	0.0001	11.8	15.75	57.1	0.063

For the included patient population receiving PRP treatment, the mean WOMAC score was 39.73 (n = 29) prior to treatment. Compared to baseline, average WOMAC scores decreased by 34.30% (μ = 25.62, p-value = 0.0002), 60.2% (μ = 15.5, p-value < 0.0001), and 58.5% (μ = 16.2, p-value = 0.0433) for patients reporting at 1-3 months, 4-6 months, and >6 months of follow-up. 

Paired analysis was also performed for each of the WOMAC subsection scores for each of the follow-up surveys in comparison to the baseline survey. At the first follow-up appointment, there was a statistically significant difference in reported pain (μ = 5.10, p-value = 0.0004), stiffness (μ = 2.45, p-value = 0.0083), and function (μ = 18.07, p-value = 0.0002) scores in comparison to baseline. This correlated with 35.7%, 31.1%, 34.3% score reductions from baseline, respectively. At the second follow-up appointment, there was a statistically significant difference in reported pain (μ = 2.44, p-value = 0.0001), stiffness (μ = 1.67, p-value = 0.0027), and function (μ = 11.39, p-value = 0.0001) scores in comparison to baseline. This correlated with 69.2%, 53.1%, 58.6% score reductions from baseline, respectively. At the third follow-up appointment, there was a statistically significant difference in reported pain score (μ = 2, p-value = 0.00086), but an insignificant difference in stiffness (μ = 2.4, p-value = 0.065), and function (μ = 11.8, p-value = 0.063) scores in comparison to baseline. This correlated with 74.8%, 32.4%, 57.1% score reductions from baseline, respectively. There was no statistically significant difference between treatment groups at any point during follow-up. 

## Discussion

Current literature regarding the use of PRP injections to treat OA of the knee is complicated by the lack of standardization among treatment protocols as well as conflicting evidence regarding their efficacy. Although limited in scope, this study corroborates the efficacy of these orthobiologic injections with sustained improvements in functional outcomes over the course of several months. In comparison to baseline, WOMAC scores were reduced by 74.8%, 32.4%, 57.1% score reductions at 3, 6, and 12 months of follow-up, respectively.

Literature exploring the efficacy of PRP for knee OA has mixed results. One randomized controlled trial (RCT) from 2013 directly compared the efficacy of PRP injections to placebo in patients with bilateral knee OA9. Both experimental groups in this study were found to have statistically significant improvements in WOMAC within two to three weeks and maintained a statistically significant improvement from baseline at six months follow-up. The mean total WOMAC scores in group A displayed a 50% decrease from baseline at six weeks follow-up, a 60% decrease at three months follow-up, and a 51% decrease at six-month follow-up. These results were similar in group B, with average total WOMAC score decreases of 55%, 54%, and 43% at first, second, and third follow-up, respectively. Similar results were described by Kon et al. [19] in which maximum benefit was reported at initial follow-up visits with a tapering of the improvement at a six-month follow-up. It is unclear if this waning effect is due to the disease progression or fading orthobiologic efficacy over time. This study maintains an increased length of follow-up, with an overall 60.2% and 58.5% decrease in WOMAC scores compared to baseline for patients following up at an average of 5.8 and 10.3 months, respectively. The results of our current study indicate that improvements in functional outcomes may be maintained over time with the use of serial injections of orthobiologic agents.

Halpern et al. reported a case series of 22 patients receiving a single injection of PRP reported WOMAC scores at 6- and 12-month follow-up appointments [20]. At 6- and 12-months follow-up, total WOMAC scores decreased by 45.10% and 56.20% compared to baseline, respectively (n = 18). This same series of patients had 41.70% and 55.90% decreases in WOMAC pain scores as well as 43.20% and 60.10% decrease in WOMAC stiffness scores at 6 and 12 months, respectively. Our PRP cohort had a greater reduction in total WOMAC scores compared to baseline at 5.8 and 10.3 months, which may be attributed to the serial injections administered at follow-up appointments. However, the most significant difference in results compared to the series of patients reported by Halpern et al. may be the decrease in the WOMAC pain scores compared to baseline at an average follow-up on 10.3 months in our PRP cohort, which was 74.8% compared to the 55.90% decrease reported by Halpern et al. at 12 months follow-up. Definitive conclusions regarding the impact of serial injections on WOMAC subsection scores are difficult to ascertain, however, given the low power of our study at an average of 10.3 months follow-up (n = 5). Another systematic review of three overlapping meta-analyses examined the functional results of PRP injections [10]. All studies found that PRP injections improved WOMAC scores for up to 12 months, although the authors reported that the current evidence does not show a clear benefit of multiple injections compared to a one-time treatment. Our current study shows promising results for the use of serial injections to treat knee OA, although further research is warranted to determine the efficacy of serial injections of PRP versus a one-time treatment. Limitations of this study include being a limited case series, a lack of a conservatively managed control group, as well as a potential for bias in data handling. 

The strengths of this study include a uniform treatment and PRP preparation protocol. The variability in preparation protocols has been a point of controversy in prior studies examining PRP. Additionally, this study has multiple limitations. Many patients were lost to follow-up over the course of the treatment schedule. This attrition was either due to loss of true patient follow-up, or the election of patients to proceed with other medical interventions in the treatment of their disease. However, these data are not currently available to the study authors. Additionally, this study does not meet the necessary population sizes to meet the power of 0.80. As this study is retrospective, it lacks a control group or any other treatment arm and it is, therefore, difficult to compare the effect size of this treatment with high fidelity.

## Conclusions

This study suggests that PRP injections may play a role in improving functional outcomes in patients with Kellgren-Lawrence grade 3 or 4 knee OA. Further studies are warranted to investigate the long-term efficacy of various orthobiologic injectables in the treatment of degenerative joint diseases. Future studies should consider consistent orthobiologic preparation protocols to ensure reproducibility.
